# Curing the pandemic of misinformation on COVID-19 mRNA vaccines through real evidence-based medicine - Part 1

**DOI:** 10.4102/jir.v5i1.71

**Published:** 2022-09-26

**Authors:** Aseem Malhotra

**Affiliations:** 1 Public Health Collaboration, London, United Kingdom

**Keywords:** COVID-19, mRNA vaccine, cardiac arrests, real evidence-based medicine, shared decision-making

## Abstract

**Background:**

In response to severe acute respiratory syndrome coronavirus 2 (SARS-CoV-2), several new pharmaceutical agents have been administered to billions of people worldwide, including the young and healthy at little risk from the virus. Considerable leeway has been afforded in terms of the pre-clinical and clinical testing of these agents, despite an entirely novel mechanism of action and concerning biodistribution characteristics.

**Aim:**

To gain a better understanding of the true benefits and potential harms of the messenger ribonucleic acid (mRNA) coronavirus disease (COVID) vaccines.

**Methods:**

A narrative review of the evidence from randomised trials and real world data of the COVID mRNA products with special emphasis on BionTech/Pfizer vaccine.

**Results:**

In the non-elderly population the “number needed to treat” to prevent a single death runs into the thousands. Re-analysis of randomised controlled trials using the messenger ribonucleic acid (mRNA) technology suggests a greater risk of serious adverse events from the vaccines than being hospitalised from COVID-19. Pharmacovigilance systems and real-world safety data, coupled with plausible mechanisms of harm, are deeply concerning, especially in relation to cardiovascular safety. Mirroring a potential signal from the Pfizer Phase 3 trial, a significant rise in cardiac arrest calls to ambulances in England was seen in 2021, with similar data emerging from Israel in the 16–39-year-old age group.

**Conclusion:**

It cannot be said that the consent to receive these agents was fully informed, as is required ethically and legally. A pause and reappraisal of global vaccination policies for COVID-19 is long overdue.

**Contribution:**

This article highlights the importance of addressing metabolic health to reduce chronic disease and that insulin resistance is also a major risk factor for poor outcomes from COVID-19.

## Vaccines save lives

The development of safe and highly effective vaccines during the latter half of the 20th century has been one of medicine’s greatest achievements. The prominent scars on my left arm are a constant reminder of the success of our ability to curb some of the deadliest diseases such as smallpox, tuberculosis (TB), measles, mumps and rubella to name but a few. Collectively, traditional vaccines are estimated to save approximately 4–5 million lives per year.^1^ The greatest success of vaccination was the global eradication of smallpox, which had a 30% mortality rate.^2^

In other words, almost one in three people who contracted it died. The development of a safe and effective vaccine after much trial and error resulted in 95 out of 100 individuals being protected from symptomatic infection from smallpox with immunity lasting five years, which by the 1970s resulted in complete eradication of the virus. Similarly, one dose of the measles vaccine is said to be ‘95% effective’. What is meant by this? What most people would assume is that 95 out of 100 who take the inoculation are protected from symptomatic infection, transmission and also have long-lasting immunity. Similarly, if exposed to chickenpox, only five out of 100 vaccinated children will catch it.

Vaccines are also some of the safest interventions in the world when compared to most drugs used in chronic disease management, as indeed we should expect, given that they are being administered to prevent something in healthy people, not treat an illness. It was therefore welcome news that in the summer of 2020, several drug companies including both Pfizer and Moderna announced the results of their 2-month randomised controlled trial that they had developed a vaccine with more than ‘95% effectiveness’ at preventing infection from what at the time was the predominantly circulating strain of the coronavirus disease 2019 (COVID-19).

## A doctor’s experience

Volunteering in a vaccine centre, I was one of the first to receive two doses of Pfizer’s messenger ribonucleic acid (mRNA) vaccine, at the end of January 2021. Although I knew my individual risk was small from COVID-19 at age 43 with optimal metabolic health, the main reason I took the jab was to prevent transmission of the virus to my vulnerable patients. During early 2021, I was both surprised and concerned by a number of my vaccine-hesitant patients and people in my social network who were asking me to comment on what I regarded at the time as merely ‘anti-vax’ propaganda.

I was asked to appear on *Good Morning Britain* after a previously vaccine-hesitant film director Gurinder Chadha, Order of the British Empire (OBE), who was also interviewed, explained that I convinced her to take the jab.

But a very unexpected and extremely harrowing personal tragedy was to happen a few months later that would be the start of my own journey into what would ultimately prove to be a revelatory and eye-opening experience so profound that after six months of critically appraising the data myself, speaking to eminent scientists involved in COVID-19 research, vaccine safety and development, and two investigative medical journalists, I have slowly and reluctantly concluded that contrary to my own initial dogmatic beliefs, Pfizer’s mRNA vaccine is far from being as safe and effective as we first thought. This critical appraisal is based upon the analytical framework for practicing and teaching evidence-based medicine, specifically utilising individual clinical expertise and/or experience with use of the best available evidence and taking into consideration patient preferences and values.

## A case study

Case studies are a useful way of conveying complex clinical information and can elicit useful data that would be lost or not be made apparent in the summary results of a clinical trial.

On 26 July 2021, my father, Dr Kailash Chand OBE, former deputy chair of the British Medical Association (BMA) and its honorary vice president (who had also taken both doses of the Pfizer mRNA vaccine six months earlier) suffered a cardiac arrest at home after experiencing chest pain. A subsequent inquiry revealed that a significant ambulance delay likely contributed to his death.^3^ But his post-mortem findings are what I found particularly shocking and inexplicable. Two of his three major arteries had severe blockages: 90% blockage in his left anterior descending artery and a 75% blockage in his right coronary. Given that he was an extremely fit and active 73-year-old man, having walked an average of 10–15 000 steps/day during the whole of lockdown, this was a shock to everyone who knew him, but most of all to me. I knew his medical history and lifestyle habits in great detail. My father who had been a keen sportsman all his life, was fitter than the overwhelming majority of men his age. Since the previous heart scans (a few years earlier, which had revealed no significant problems with perfect blood flow throughout his arteries and only mild furring), he had quit sugar, lost belly fat, reduced the dose of his blood pressure pills, started regular meditation, reversed his prediabetes and even massively dropped his blood triglycerides, significantly improving his cholesterol profile.

I couldn’t explain his post-mortem findings, especially as there was no evidence of an actual heart attack but with severe blockages. This was precisely my own special area of research. That is, how to delay progression of heart disease and even potentially reverse it. In fact, in my own clinic, I successfully prescribe a lifestyle protocol to my patients on the best available evidence on how to achieve this. I’ve even co-authored a high-impact peer-reviewed paper with two internationally reputed cardiologists (both editors of medical journals) on shifting the paradigm on how to most effectively prevent heart disease through lifestyle changes.^4^ We emphasised the fact that coronary artery disease is a chronic inflammatory condition that is exacerbated by insulin resistance. Then, in November 2021, I was made aware of a peer-reviewed abstract published in *Circulation*, with concerning findings. In over 500 middle-aged patients under regular follow up, using a predictive score model based on inflammatory markers that are strongly correlated with risk of heart attack, the mRNA vaccine was associated with significantly increasing the risk of a coronary event within five years from 11% pre-mRNA vaccine to 25% 2–10 weeks post mRNA vaccine. An early and relevant criticism of the validity of the findings was that there was no control group, but nevertheless, even if partially correct, that would mean that there would be a large acceleration in progression of coronary artery disease, and more importantly heart attack risk, within months of taking the jab.^5^ I wondered whether my father’s Pfizer vaccination, which he received six months earlier, could have contributed to his unexplained premature death and so I began to critically appraise the data.

## Questioning the data

I recalled a cardiologist colleague of mine informing me, to my astonishment at the time, that he had made a decision not to take the vaccine for a number of reasons, including his personal low background COVID-19 risk (see Table 1)^6^ and concerns regarding unknown short- and longer-term harms. One thing that alarmed him about Pfizer’s pivotal mRNA trial published in *The New England Journal of Medicine* was the data in the supplementary appendix, specifically that there were four cardiac arrests in those who took the vaccine versus only one in the placebo group.^7^ These figures were small in absolute terms and did not reach statistical significance in the trial, suggesting that it may just be coincidence, but without further studies it was not possible to rule out this being a genuinely causal relationship (especially without access to the raw data), in which case it could have the effect of causing a surge in cardiac arrests once the vaccine was rolled out to tens of millions of people across the globe.

**TABLE 1:** Infection fatality rate of ancestral variants of COVID-19 pre-vaccination by age.

Age	Median IFR %	Median IFR (absolute)	Survival rate estimate (%)
0–19	0.0027	1 in 37 037	99.9973
20–29	0.0140	1 in 7143	99.9860
30–39	0.0310	1 in 3225	99.9690
40–49	0.0820	1 in 1220	99.9180
50–59	0.2700	1 in 370	99.7300
60–69	0.5900	1 in 169	99.4100
> 70 community	2.4000	1 in 42	97.6000
> 70 overall	5.5000	1 in 18	94.5000

*Source:* Adapted from Axfors C, Ioannidis JPA. Infection fatality rate of COVID-19 in community-dwelling elderly populations. Eur J Epidemiol. In press 2022;37(3):235–249. https://doi.org/10.1007/s10654-022-00853-w

IFR, infection fatality rate.

In terms of efficacy, headlines around the world made very bold claims of 95% effectiveness, the interchangeable use of ‘efficacy’ and ‘effectiveness’ glossing over the big difference between controlled trial and real-world conditions.^8^ It would be understandable for the lay public and doctors to interpret this that if 100 people are vaccinated then 95% of people would be protected from getting the infection. Even the Centers of Disease Control (CDC) director Rochelle Walensky recently admitted in an interview that it was initial news from CNN that made her optimistic that the vaccine would significantly stop transmission and infection, but this was later to be proved far from true for the COVID-19 vaccines.^9^ The original trial revealed that a person was 95% ‘less likely’ to catch the autumn 2020 variant of COVID-19. This is known in medical speak as relative risk reduction, but to know the true value of any treatment one needs to understand for that person, by how much is their individual risk reduced by the intervention – that is, the *absolute individual risk reduction*.

Importantly, it turns out that the trial results suggest that the vaccine was only preventing a person from having a symptomatic positive test, and the absolute risk reduction for this was 0.84% (0.88% reduced to 0.04%). In other words, if 10 000 people had been vaccinated and 10 000 had not, for every 10 000 people vaccinated in trial 4 would have tested positive with symptoms compared to 88 who were unvaccinated. Even in the unvaccinated group, 9912 of the 10 000 (over 99%) would not have tested positive during the trial period. Another way of expressing this is that you would need to vaccinate 119 people to prevent one such symptomatic positive test (assumed to be indicative of an infection, which, in itself, is potentially misleading but beyond the scope of this article).^10^

This absolute risk reduction figure (0.84%) is extremely important for doctors and patients to know but how many of them were told this when they received the shot? Transparent communication of risk and benefit of any intervention is a core principle of ethical evidence-based medical practice and informed consent.^11^

The Academy of Medical Royal Colleges made this clear in a paper published in the *BMJ* in 2015.^12^ A co-author at the time was also the then chair of the General Medical Council. In fact, in a 2009 World Health Organization (WHO) bulletin Gerd Gigerenzer, the director of the Max Planck institute stated, ‘It’s an ethical imperative that every doctor and patient understand the difference between relative and absolute risks to protect patients against unnecessary anxiety and manipulation’.^13^

Contrary to popular belief, what the trial did not show was any statistically significant reduction in serious illness or COVID-19 mortality from the vaccine over the 6-month period of the trial, but the actual numbers of deaths (attributed to COVID-19) are still important to note. There were only two deaths from COVID-19 in the placebo group and one death from COVID-19 in the vaccine group. Looking at all-cause mortality over a longer period, there were actually slightly more deaths^14^ in the vaccine group (19 deaths) than in the placebo group (17 deaths). Also of note was the extremely low rate of COVID-19 illness classed as severe in the placebo group (nine severe cases out of 21 686 subjects, 0.04%), reflecting a very low risk of severe illness even in regions chosen for the trial because of perceived high prevalence of infection.

Finally, the trials in children did not even show a reduction in symptomatic infections but instead used the surrogate measure of antibody levels in the blood to define efficacy, even though the relationship between Wuhan-spike vaccine-induced antibody levels and protection from infection is tenuous, at best. The Food and Drug Administration’s (FDAs) own website states that:

[*R*]esults from currently authorised SARS-COV-2 antibody tests should not be used to evaluate a person’s level of immunity or protection from COVID-19 at any time, and especially after the person received a COVID-19 vaccination.^15^

Now that we know what the published trial did and did not show in terms of the vaccine efficacy, we can attempt to extrapolate what the effect of the vaccine would be in reducing mortality or any other adverse outcome from the virus. If there is a 1 in 119 chance the vaccine protects you from getting symptomatic infection from ancestral variants, then to find the protection against death, this figure (*n* = 119) must be multiplied by the number of infections that lead to a single death for each age group. This would give (for up to two months after the inoculation) the absolute risk reduction (for death) from the vaccine. For example, if my risk at age 44 from dying from Delta (should I get infected with it) is 1 in 3000, then the absolute risk reduction from the vaccine protecting me from death is 1 over 3000 multiplied by 119, that is, 1 per 357 000.

**FIGURE 1:**
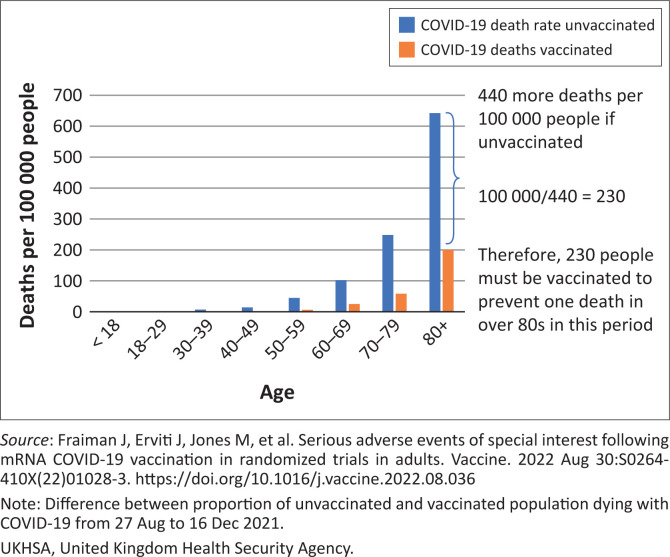
Calculation of number needed to be vaccinated from COVID-19 death rates in vaccinated and unvaccinated from UKHSA data for England during the Delta wave. The difference between the deaths that occurred in the vaccinated and that would have occurred if they had the same rate as the unvaccinated was used to calculate the number of people who would need to be vaccinated to prevent a single death.

Of course, even for those people who do become infected the vaccination may provide some protection against death. From observational data it is possible to calculate the number who would need to be vaccinated to prevent a COVID-19 death. For example, comparing the population death rates^16^ during the Delta wave gives 230 for people over 80s needing to be vaccinated to prevent a single death in that period with that number rising to 520 for people in their 70s and 10 000 for people in their 40s (see Table 2 and Figure 1^17^). However, these figures will be distorted by inaccuracies in the measure of the size of the unvaccinated population. As also pointed out in a recent editorial by John Ioannidis in *BMJ* evidence-based medicine the inferred efficacy of the vaccine from non-randomised studies may be ‘spurious’, with bias being generated by ‘pre-existing immunity, vaccination misclassification, exposure differences, testing, disease risk factor confounding, hospital admission decision, treatment use differences and death attribution’.^18^

**TABLE 2:** Deaths prevented, and number needed to vaccinate to prevent a death based on death rates and case fatality rates from UKHSA data for England during Delta wave.

Age	Deaths prevented (in England) based on differences in death rates per 100 000	Number needed to vaccinate per death prevented based on differences in death rates per 100 000
< 18	−0.1	Negative
18–29	70	93 000
30–39	240	27 000
40–49	640	10 000
50–59	2740	2600
60–69	4580	1300
70–79	9100	520
80+	11 900	230

**Total**	**29 270**	**-**

*Source:* Adapted from HART. How many injections to prevent one covid death? [homepage on the Internet]. No date. Available from: https://www.hartgroup.org/number-needed-to-vaccinate/

UKHSA, United Kingdom Health Security Agency.

These numbers are for the whole population of England and do not necessarily apply to the healthy; more than 95% of deaths were in people with pre-existing conditions.^19^ It is also important to note that the vaccinated and unvaccinated populations are different in other ways, which could bias the death data. For example, the unvaccinated are more likely to be from a lower socioeconomic demographic, which puts them at a greater risk of severe illness or death should they be infected.

Professor Carl Heneghan, the director of the Centre of Evidence Based Medicine in Oxford, has explained his own clinical experience of healthy user bias. Some of his own patients who ended up in intensive care unit (ICU) with COVID-19 (classified as unvaccinated) did not take the vaccine because they were already suffering from terminal illness.

Given these limitations, the above figures are likely an overestimate of the individual benefit of vaccination; the open and frank discussion of such uncertainties is an essential component of shared decision-making.

What should be part of the shared decision-making informed consent discussion when any member of the public is considering taking the shot is something along these lines: Depending on your age, several hundreds or thousands of people like you would need to be injected in order to prevent one person from dying from the Delta variant of COVID-19 over a period of around three months. For the over 80s, this figure is at least 230, but it rises the younger you are, reaching at least 2600 for people in their 50s, 10 000 for those in their 40s, and 93 000 for those between 18 and 29 years. For omicron, which has been shown to be 30% – 50% less lethal, meaning significantly more people would need to be vaccinated to prevent one death. How long any protection actually lasts for is unknown; boosters are currently being recommended after as short a period as 4 months in some countries.

But how many people have had a conversation that even approaches an explanation similar to that? This is before we get into the known, unknown and as yet to be fully quantified harms.

Although many have proposed that omicron is intrinsically less lethal (supported by observed molecular differences between omicron and the Wuhan-type virus) immunity built up by prior exposure protecting against severe illness is likely to be relevant to some extent as well. The critical point to note that, whether it is a viral or immune-related phenomenon, the milder nature of omicron is evident in the unvaccinated and therefore the reduction in mortality should not be attributed to vaccines. ≤

## What are the harms?

Concerns have already been raised about the under-reporting of adverse events in the clinical trials for the COVID-19 vaccines. Investigative medical reporter Maryanne Demasi analysed the various ways that the pivotal mRNA trials failed to account for serious harms.^20^ Not only were trial participants limited to the type of adverse event they could report on their digital apps, but some participants who were hospitalised after inoculation were withdrawn from the trial and not reported in the final results. After two months into the pivotal trials, the FDA allowed vaccine companies to offer the vaccine to subjects in the placebo group, essentially torpedoing any chance of properly recording adverse events from that point on, forcing a reliance of pharmacovigilance data.

Such data have shown that one of the most common mRNA COVID-19 vaccine-induced harms is myocarditis. A study across several Nordic countries showed an increased risk from mRNA vaccination over background, especially in young males.^21^ Authorities have repeatedly maintained that myocarditis is more common after COVID-19 infection than after vaccination.^22^ However, trial data demonstrating that vaccination reduces the risk of myocarditis in subsequent infection is elusive, and in fact the risks may be additive. Incidence of myocarditis rocketed from spring 2021 when vaccines were rolled out to the younger cohorts having remained within normal levels for the full year prior, despite COVID-19,^23^ with the most up-to-date evidence, a paper from Israel^24^ found that the infection itself, prior to roll-out of the vaccine, conferred no increase in the risks of either myocarditis or pericarditis from COVID-19, strongly suggesting that the increases observed in earlier studies were because of the mRNA vaccines, with or without COVID-19 infections as an additional risk in the vaccinated.^24^

Indeed, this reflects my own clinical experience of advising and managing several patients in the community who presented with a clear suggestion from the history of myocarditis post mRNA vaccination but aren’t necessarily unwell enough to require hospital admission. A very fit lady in her 50s developed fatigue and shortness of breath on exertion a few weeks after her second Pfizer injection. An echocardiogram revealed severe impairment of her left ventricular function. Another lady in her 30s experienced similar symptoms with distressing palpitations within a few days of her second shot; mild left ventricular impairment was also present on echo and a subsequent cardiac MRI scan revealed several areas of *late gadolinium enhancement*, a feature seen on the scan, which is consistent with damaged heart tissue, and given that heart cells cannot be replaced this is likely to have a long-term impact.

Although vaccine-induced myocarditis is not often fatal in young adults, MRI scans reveal that, of the ones admitted to hospital, approximately 80% have some degree of myocardial damage.^25,26^ It is like suffering a small heart attack and sustaining some – likely permanent – heart muscle injury. It is uncertain how this will play out in the longer-term, including if, and to what degree, it will increase the risk of poor quality of life or potentially more serious heart rhythm disturbances in the future.

A number of reports have produced concerning rates of myocarditis, depending on age, ranging from 1 in 6000 in Israel^27^ to 1 in 2700 in a Hong Kong study in male children and adolescents aged 12–17 years.^28^ Most of the epidemiology studies that have been carried out have measured myocarditis cases that have been diagnosed in a hospital setting, and do not claim to be a comprehensive measure of more mild cases (from which long-term harm cannot be ruled out). In addition, under-reporting of adverse events is the scourge of pharmacovigilance data.^29^

The United Kingdom relies on the Medicines and Health Regulatory Agency’s (MHRAs) ‘Yellow Card’ reporting system,^30^ which is far from adequate to cope with a rapid roll-out of a brand new product. It only detected the clotting problems that resulted in the withdrawal of the AstraZeneca product in April 2021 for younger people after 9.7 million doses had been given in the United Kingdom^31^; in contrast, Denmark detected the problem after only 150 000 doses had been administered.^32^

In the United Kingdom, since the vaccine roll-out there have been almost 500 000 adverse event reports recorded (via the Yellow Card system) in association with the mRNA COVID-19 vaccinations involving over 150 000 individuals. In terms of the number of reports per person (i.e. having received at least one dose), the MHRA figures show around 1 in 120 suffering a likely adverse event that is beyond mild.^30^ However, the MHRA are unclear about the rate and furthermore do not separate out the serious adverse events. Nevertheless, this level of reporting is unprecedented in the modern medical era and equals the total number of reports received in the first 40 years of the Yellow Card reporting system (for all medicines – not just vaccines) up to 2020.^33^ In comparison, for the measles, mumps and rubella (MMR) vaccine, the number of reports per person vaccinated was around 1 in 4000, more than thirty times less frequent than the 1 in 120 Yellow Card reports for COVID-19 vaccine recipients.^34^ Norway does separate out the reported serious adverse reactions and has shown a rate of approximately 1 in 1000 after two doses of BioNTech/Pfizer mRNA product that result in hospitalisation or are life changing.^35^

Another, and more useful, source of information (because of the level of detail for each report made available to the public) is the United States (US) Vaccine Adverse Effect Reporting System (VAERS). As with the UK’s system, the level of reports – including serious ones – associated with COVID-19 vaccines is completely unprecedented. For example, over 24 000 deaths have now been recorded in VAERS as of 02 March 2022; 29% of these occurred within 48 h of injection, and half within two weeks. The average reporting rate prior to 2020 was less than 300 deaths per annum. One explanation often given for this is that the COVID-19 vaccine roll-out is unprecedented in scope; however, this is not valid, since (for the last decade at any rate) the United States has administered 150 million – 200 million vaccinations annually. Another criticism of VAERS is that ‘anyone can make an entry’, yet, in fact, an analysis of a sample of 250 early deaths suggested that the vast majority are hospital or physician entries,^36^ and knowingly filing a false VAERS report is a violation of Federal law punishable by fine and imprisonment.^37^

Given that VAERS was set up to generate early signals of potential harm for new vaccines, and was instrumental in doing so for several products, it seems perverse to only now criticise it as unreliable when there seem to have been no changes in the way it operates.

It has been estimated that serious adverse effects that are officially reported are actually a gross underestimate, and this should be borne in mind when the above comments in relation to VAERS reports are considered. For example, a paper by David Kessler (a former FDA Commissioner) cites data suggesting that as few as 1% of serious adverse events are reported to the FDA.^38^ Similarly in relation to the Yellow Card scheme in the United Kingdom, it has been estimated that only 10% of serious adverse effects are reported.^39,40^ A recent pre-print publication co-authored by some of the most trusted medical scientists in the world in relation to data transparency adds validity to pharmacovigilance data. Accessing data from the FDA and health Canada websites and combining results from journal articles that published the Pfizer and Moderna trials, the authors concluded that the absolute risk of a serious adverse event from the mRNA vaccines (a rate of one in 800) significantly exceeded the risk of COVID-19 hospitalisation in randomised controlled trials.^17^

What VAERS and other reporting systems (including the yet to be accessed and independently evaluated raw data from randomised controlled trials) will miss are potential medium to longer term harms that neither patients nor doctors will automatically attribute to the drug. For example, if the mRNA vaccine increases the risk of a coronary event within a few months (in what was a likely contributory factor in my father’s sudden cardiac death), then this would increase event rates well beyond the first few weeks of the jab yet linking it back to the vaccine, and thus reporting it is highly unlikely to occur later on.

It is instructive to note that according to ambulance service data, in 2021 (the year of the vaccine roll-out), there were approximately an extra 20 000 (~20% increase) out-of-hospital cardiac arrest calls compared to 2019, and approximately 14 000 more than in 2020. Data obtained under Freedom of Information laws from one of the largest ambulance trusts in England suggest that there was no increase from November 2020 to March 2021, and thereafter the rise has been seen disproportionately in the young.^41^ This is a huge signal that surely needs investigating with some urgency.^42^

Similarly, a recent paper in *Nature* revealed a 25% increase in both acute coronary syndrome and cardiac arrest calls in the 16- to 39-year-old age groups significantly associated with administration with the first and second doses of the mRNA vaccines but no association with COVID-19 infection.^43^ The authors state that:

[*T*]he findings raise concerns regarding vaccine-induced undetected severe cardiovascular side effects and underscore the already established causal relationship between vaccines and myocarditis, a frequent cause of unexpected cardiac arrest in young individuals. (p. 1)

The disturbing findings in this paper have resulted in calls for a retraction. In the past, scientists with a different view of how data should be analysed would have published a paper with differing assumptions and interpretation for discussion. Now they try to censor.

Many other concerns have been raised about potential harms from the vaccines in the mid- to long-term. Although some of these concerns remain hypothetical, it may be a grave mistake to focus only on what can be measured and not on the wider picture, especially for the young.

## What could be the mechanism of harm?

**FIGURE 2:**
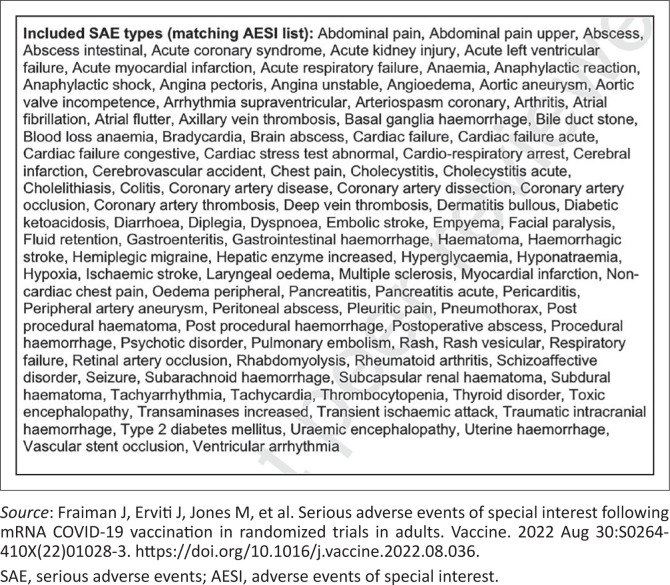
World Health Organization endorsed a list of adverse events of special interest associated with COVID-19 vaccinations.

For ‘conventional vaccines’, an inert part of the bacteria or virus is used to ‘educate’ the immune system. The immune stimulus is limited, localised and short-lived. For the COVID-19 vaccines, spike protein has been shown to be produced continuously (and in unpredictable amounts) for at least four months after vaccination^44^ and is distributed throughout the body after intramuscular injection.^45^ For the severe acute respiratory syndrome coronavirus 2 (SARS-CoV-2) vaccines, the spike protein was chosen, possibly because it enables cell entry. However, this protein is not inert, but rather it is the source of much of the pathology associated with severe COVID-19, including endothelial damage,^46^ clotting abnormalities^47^ and lung damage. It is instructive to note that prior to roll-out of the mRNA products, the WHO endorsed a priority list of potential serious adverse events of special interest that may occur as a direct result of COVID-19 vaccines. The list was based upon the specific vaccine platform, adverse events associated with prior vaccines in general, theoretical associations based upon animal models and COVID-19-specific immunopathogenesis^40^ (see Figure 2).

## Is the vaccine doing more harm than good?

The most objective determinant of whether the benefits of the vaccines outweigh the harms is by analysing its effects on ‘all-cause mortality’. This gets round the thorny issue as to what should be classified as a COVID-19 death, and also takes full account of any negative effects of the vaccine. It would be surprising – to say the least – if during an apparently deadly pandemic, an effective vaccine could not clearly and unequivocally be shown to reduce all-cause mortality.

Pfizer’s pivotal mRNA trial in adults did not show any statistically significant reduction in all-cause mortality, and in absolute terms there were actually slightly more deaths in the treatment arm versus in the placebo.

Work by Fenton et al. showed an unusual spike in mortality in each age group of the unvaccinated population, which coincides with the vaccine roll-out for each age group.^48^ The rapid shrinking in the size of this population means a small-time lag could theoretically produce this effect artifactually. Alternative explanations must include the (more likely) possibility that a rise in mortality after vaccination was misattributed to the unvaccinated population: in other words, those counted as ‘unvaccinated deaths’ would in fact be those who had died within 14 days of being vaccinated (a freedom of information [FOI] request has now confirmed that authorities in Sweden were indeed categorising deaths within 14 days of dosing as unvaccinated, creating a misleading picture of efficacy vs death).

One has to raise the possibility that the excess cardiac arrests and continuing pressures on hospitals in 2021/2022 from non-COVID-19 admissions may all be signalling a non-COVID-19 health crisis exacerbated by interventions, which would of course also include lockdowns and/or vaccines.

Given these observations, and reappraisal of the randomised controlled trial data of mRNA products, it seems difficult to argue that the vaccine roll-out has been net beneficial in all age groups. While a case can be made that the vaccines may have saved some lives in the elderly or otherwise vulnerable groups, that case seems tenuous at best in other sections of the population, and when the possible short-, medium- and unknown longer-term harms are considered (especially for multiple injections, robust safety data for which simply does not exist), the roll-out into the entire population seems, at best, a reckless gamble. It’s important to acknowledge that the risks of adverse events from the vaccine remain constant, whereas the benefits reduce over time, as new variants are (1) less virulent and (2) not targeted by an outdated product. Having appraised the data, it remains a real possibility that my father’s sudden cardiac death was related to the vaccine. A pause and reappraisal of vaccination Policies for COVID-19 is long overdue.
